# Bi-component synergic effect in lily-like CdS/Cu_7_S_4_ QDs for dye degradation†

**DOI:** 10.1039/c8ra09331h

**Published:** 2019-01-18

**Authors:** Mengli Wan, Shizhong Cui, Wutao Wei, Siwen Cui, Kongyao Chen, Weihua Chen, Liwei Mi

**Affiliations:** Center for Advanced Materials Research, Zhongyuan University of Technology Zhengzhou 450007 China mlwzzu@163.com; College of Chemistry and Molecular Engineering, Zhengzhou University Zhengzhou 450001 China chenweih@zzu.edu.cn

## Abstract

CdS has attracted extensive attention in the photocatalytic degradation of wastewater due to its relatively narrow bandgap and various microstructures. Previous reports have focused on CdS coupled with other semiconductors to reduce the photocorrosion and improve the photocatalytic performance. Herein, a 3D hierarchical CdS/Cu_7_S_4_ nanostructure was synthesized by cation exchange using lily-like CdS as template. The heterojunction material completely inherits the special skeleton of the template material and optimizes the nano-scale morphology, and achieves the transformation from nanometer structure to quantum dots (QDs). The introduction of Cu ions not only tuned the band gap of the composites to promote the utilization of solar photons, more importantly, Fenton-like catalysis was combined into the degradation process. Compared with the experiments of organic dye degradation under different illumination conditions, the degradability of the CdS/Cu_7_S_4_ QDs is greatly superior to pure CdS. Therefore, the constructed CdS/Cu_7_S_4_ QDs further realized the optimization of degradation performance by the synergic effect of photo-catalysis and Fenton-like catalysis.

## Abstract 2

CdS has attracted extensive attention in the photocatalytic degradation of wastewater due to its relatively narrow bandgap and various microstructures.

## Graphical abstract

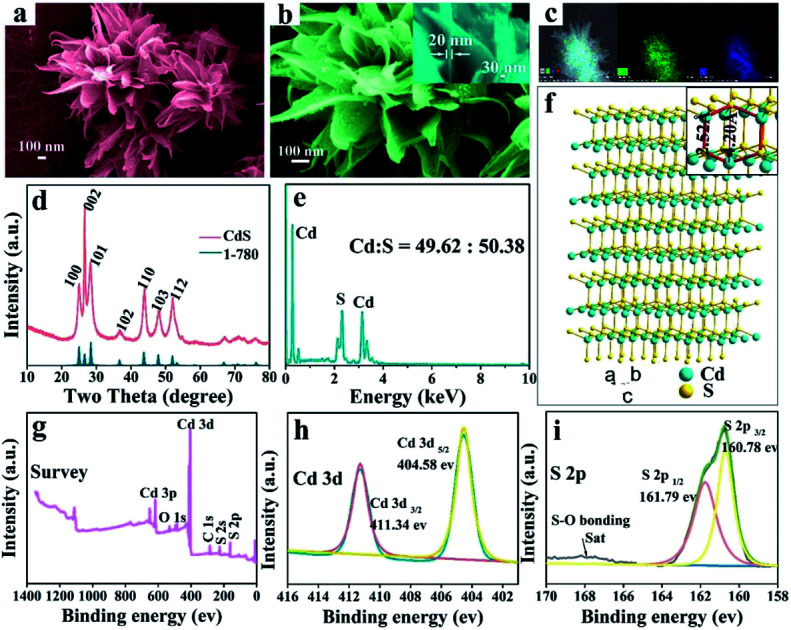

## Introduction

Industrial wastewater, especially dye wastewater containing refractory and virulent organic ingredients, is one of the main culprits of ecological environmental damage.^1,2^ The current general strategies for treating dye wastewater are microbiological decomposition, physical absorption and photocatalyst degradation.^3–5^ However, rigorous treatment systems, incomplete degradation resulting in secondary pollution and low light utilization greatly limit their large-scale application. Compared with traditional methods, the Fenton oxidation process is based on freshly generated hydroxyl radicals (OH˙) with a high oxidation potential (2.8 V) to completely break down organic macromolecules into small molecules (CO_2_, H_2_O, *et al.*) and have become one of the highly efficient candidates for degradation of dye wastewater, which relies heavily on the activity of Fenton catalysts.^6,7^ Nevertheless, the traditional Fe-based Fenton catalysts are harsh on the pH of the degradation system, easily oxidized and difficult to preserve.^8,9^ To push the complete treatment of dye wastewater, it is highly desirable to develop a Fenton-like catalyst with adequate activity and stability.^6,10^

Copper sulfide is considered to be an ideal candidate for Fenton-like catalysis because of its multivalent state similar to Fe-based catalysts and high stability and low dependence on degradation system. The performance of the material is closely related to their structure and morphology.^11,12^ So the development of a novel copper sulfide crystal with a three-dimensional hierarchical structure as a high efficiency Fenton-like catalyst is of great interest. In recent years, the micro/nano construction method is often used to construct inorganic compounds with a special structure and morphology for providing a large specific surface area and abundant active sites.^13–15^ For instance, solvothermal synthesis of 3D hierarchical CuS microspheres and CuS nanoparticles were prepared by *in situ* precipitation.^16,17^ Besides, the soft-template method is also a popular method used to regulate the morphology of copper sulfide. Different morphologies (snowflake-like patterns, flower-like and porous hollow microspheres) of CuS nanostructures were synthesized by adjusting the amount of biomolecule assistance.^18^ And a CuS nanoplate was prepared in the presence of cation surfactant cetyltrimethylammonium bromide (CTAB).^19^ These reports have promoted the rapid development of morphology research of copper sulfide. There are several classical reports on regulation of morphology and structure of copper sulfide at the lattice level.^20–23^ These reports provide a good reference for morphological control. Recently, ion-exchange method is considered to be a general strategy for the preparation of materials with special structures and morphologies, which uses an existing crystal lattice as a template to enable the realization of morphological inheritance of parent materials and introduce abundant lattice defects resulting in the synergy and complementarity between different atoms. For example, lavender-like Ni_3_S_2_/Co_9_S_8_/NiSe nanoarrays synthesized by sequential partial ion exchange exhibited excellent rate performance as electrode materials for supercapacitors.^20^ The ions exchange between bromide or iodide ions and chloride ions on the QDs improve photoluminescence quantum yield.^21^ Therefore, it is of great significance to prepare copper sulfide with excellent morphology and excellent Fenton-like catalyst activity by ion exchange method by using the parent material with special structure and morphology.

As a typical photocatalyst, the fabrication of CdS nanocrystals with various structures, including QDs, nanospheres, nanorods and nanoflowers has been extensively studied,^30–33^ especially the CdS QDs owing to their unique optoelectronic properties including a high absorption coefficient, band gap tunability and potential multiple exciton generation.^34,35^ Micro-emulsion method and hot injection method as the common strategies for synthesizing CdS QDs have cumbersome process.^26–29^ The design of a mild and simple method to realize the transformation of CdS micro–nanometer structure to CdS QDs will open a shortcut for the preparation of CdS QDs. In previous reports,^24,25^ ion exchange method has been proved to be an ingenious strategy to realize the continuous transformation of material composition, which can also be used to replace most of cadmium ions in CdS micro–nanomaterials with multi-functional copper ions, leading to the preparation of CdS/Cu_*x*_S QDs heterojunction material with the synergistic effect of photocatalyst and Fenton-like catalyst. These findings have been rarely reported.

Herein, the 3D hierarchical lily-like CdS microflowers with several single ultrathin nanosheets as building block were successfully synthesized *via* a simple solvent-thermal method with the assistance of CTAB. This special three-dimensional hierarchical structure endows the as-obtained CdS material with large specific surface area and luxuriant active sites, which can improve the internal performance effectively. Consequently, CdS/Cu_7_S_4_ nanocrystals were synthesized through the continuous copper-ion exchange using the 3D hierarchical lily-like CdS microflowers as template. As the proportion of copper ions to replace cadmium ions in the lattice increases, the controllable preparation of 3D hierarchical lily-like CdS/Cu_7_S_4_ QDs was also achieved. On the whole, CdS/Cu_7_S_4_ perfectly inherited the excellent morphology of the parent material. Moreover, many nanoholes and lattice defects are introduced into the target material along with ion exchange, which further increases the specific surface area and active sites of nanocrystals effectively. More importantly, the heterojunction materials of CdS/Cu_7_S_4_ QDs realize the synergistic effect of photocatalyst and Fenton-like catalyst. Then, CdS/Cu_7_S_4_ QDs was used to degrade the organic dyes such as methylene blue (MB) and rhodamine B (RB) assisted by hydrogen peroxide and illuminated by ultraviolet light. Compared with CdS nanocrystals, the 3D hierarchical lily-like CdS/Cu_7_S_4_ QDs show the higher degradation efficiency. This work provides a novel reference for the preparation of CdS QDs and multi-functional and efficient dye wastewater degradation catalyst.

## Experimental section

### Chemical and reagents

All chemical reagents in the present work were analytical grade and used without further purification. Cadmium nitrate tetrahydrate (Cd(NO_3_)_2_·4H_2_O, AR, 99%, Sinopharm Chemical Reagent Co., Ltd), copper nitrate trihydrate (Cu(NO_3_)_2_·3H_2_O, AR, 99%, Sinopharm Chemical Reagent Co., Ltd), thiourea (CH_4_N_2_S, AR, 99%, Sinopharm Chemical Reagent Co., Ltd), hexadecyltrimethylammonium bromide (C_19_H_42_BrN, AR, 99%, Sinopharm Chemical Reagent Co., Ltd), ethylenediamine (C_2_H_8_N_2_, AR, 99%, Sinopharm Chemical Reagent Co., Ltd), ethylene glycol (C_2_H_6_O_2_, AR, 99%, Sinopharm Chemical Reagent Co., Ltd), ethanol (C_2_H_5_OH, AR, 99%, Sinopharm Chemical Reagent Co., Ltd) and distilled water.

### Synthesis of 3D hierarchical lily-like CdS microflowers

3D hierarchical lily-like CdS microflowers were prepared by a modified one step solvothermal method. In a typical procedure, 0.2314 g of cadmium nitrate tetrahydrate, 0.0571 g of thiourea, 0.1 g of CTAB, 9 mL of ethylene glycol and 15 mL of anhydrous ethylenediamine as the mixed solvent were successively added into a 30 mL of Teflon-lined autoclave, stirred for 8 h, maintained at 160 °C for 4 h and then cooled down to room temperature. The resulting yellow solid products were collected by centrifugation and washed with distilled water and 95% ethanol for several times. The product was then dried at 60 °C for 8 h, and collected for further characterization.

In order to study the growth mechanism of 3D hierarchical lily-like CdS microflowers using the above method, a series of samples were synthesized for different reaction time. Therefore, several parallel experiments were executed under the same conditions, except reaction time. The samples were named as CdS-1, CdS-2, and CdS-3 corresponding to 40 min, 100 min and 4 h of reaction time, respectively.

### Synthesis of CdS/Cu_7_S_4_ nanomaterials

The hierarchical CdS/Cu_7_S_4_ nanomaterials were synthesized by the ion exchange method with the as-prepared CdS microflowers as a precursor and template. The experimental conditions were same as the above CdS synthesis process. In brief, 0.5 mmol CdS nanomaterials and a certain amount of Cu(NO_3_)_2_·3H_2_O were added into the above reaction solution. The mixture was transferred into a 30 mL Teflon-lined autoclave, stirred for 0.5 h, and then reacted for overnight at room temperature about 25 °C. Finally, the product was collected by centrifugation, washed with distilled water and ethanol for several times each, and then dried at 60 °C for 8 h.

In order to explore the influence of Cu^2+^ concentration on the morphology and component of the final products during ion exchange reaction process. A series of samples were prepared with different cation mole ratio. The molar ratio of CdS and Cu^2+^ was 1 : 0.05, 1 : 0.2, 1 : 0.5, 1 : 1 and 1 : 2, and the corresponding samples were labelled as CdS/Cu_7_S_4_-1, CdS/Cu_7_S_4_-2, CdS/Cu_7_S_4_-3, CdS/Cu_7_S_4_-4 and CdS/Cu_7_S_4_-5, respectively.

### Catalytic activity evaluation

MB and RB are representative organic dyes used to evaluate the catalytic performance. Typically, the as-prepared dry sample as the catalyst (30 mg) was added to the single dye solution (30 mg L^−1^, 30 mL), and the reaction was initiated by adding H_2_O_2_ (10 mL). The reaction was conducted under different illumination environment (include UV light, visible light and in dark condition), and the whole reaction process was proceed at 30 °C under slowly stirring. Every 5 min of reaction time, about 3 mL of solution was collected from the dye solution. The mixture was centrifuged at 12 000 rpm for 2 min and the remaining solution was continuously used for analysis. The concentration of dye was determined by a UV-vis spectrophotometer. The mixed solution of RB@MB was degraded by the CdS, CdS/Cu_7_S_4_ catalysts in the same manner, the only difference was that the dyes solution consisted of MB (30 mg L^−1^, 15 mL) and RB (30 mg L^−1^, 15 mL).

### Radical-trapping experiments

In order to identify the major active radical in the degradation of MB solution, isopropyl alcohol (IPA, a hydroxyl radical scavenger), disodium ethylenediaminetetraacetate (Na_2_-EDTA, a hole scavenger) and benzoquinone (BQ, a superoxide anion radical scavenger) were used in radical trapping experiment. 1 mM scavenger, 30 mg catalyst and 10 mL H_2_O_2_ were added into 30 mL of MB solution (30 ppm). The subsequent experiment operation was the same as the degradation experiment.

### Characterization

Characterizations were carried out to fully realize the physical and chemical properties of the samples. X-ray diffraction (XRD) patterns were obtained with a Bruker D8 Advance X-ray powder diffractometer using Cu-Kα irradiation at a scan rate of 0.1° s^−1^ All XRD measurements were performed within 20° ≤ 2*θ* ≤ 80°. The X-ray photoelectron spectroscopy (XPS) of the as-prepared samples were analysed using a Kratos AXIS ULTRA X-ray photoelectron microscope with Al-Kα X-rays as the excitation source. The morphologies and sizes of the as-obtained samples were investigated with a scanning electron microscope (SEM; Zeiss EVO LS-15) equipped with an energy-dispersive X-ray spectroscopy (EDS) system. The ultraviolet-visible (UV-vis) spectra were measured on a PerkinElmer Lambda 950 UV/VIS/NIR spectrometer in the 350 nm to 800 nm wavelength range. The photoluminescence spectra of the samples were measured by Hitachi U-4100 with an excitation wavelength of 450 nm. The photocurrent intensity was carried out on CHI 660E electrochemical workstation (Chenhua Instrument, China). The photocurrent measurements were carried out in 0.2 M of Na_2_SO_4_ solution using an Ag/AgCl as the reference electrode, a Pt wire as the counter electrode and ITO precoated photocatalyst (0.25 cm^2^). Xe lamp equipped with a 420 nm monochromator was used to provide the visible light irradiation. The Mott–Schottky measurements were carried out at 10 Hz and the three-electrode system was same as the photocurrent measurements. Nitrogen adsorption/desorption measurements were performed to investigate the surface characteristics on a Micromeritics ASAP2420 instrument. The specific surface area of the as-prepared samples were calculated by the Brunauer–Emmett–Teller (BET) method.

## Results and discussion

**Fig. 1:**
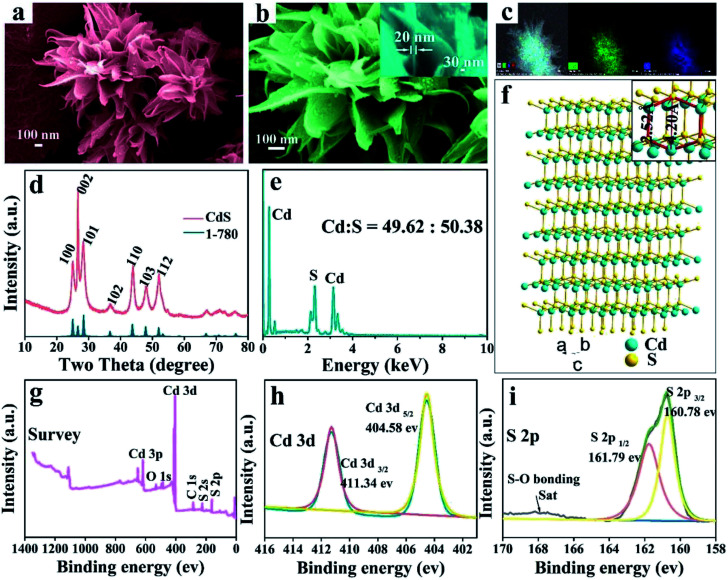
Characterization of CdS: (a) and (b) the SEM images of CdS microflowers and the high-magnification SEM image of single nanosheet (inset), (c) the EDS surface scanning images, (d) XRD pattern, (e) the EDS spectrum and (f) the crystalline structure. XPS spectra showing (g) the survey spectrum, (h) Cd 3d and (i) S 2p peak regions.

3D hierarchical CdS nanomaterials were prepared by a modified one step solvothermal method. The high-magnification SEM images (Fig. 1a and b) reveal that the as-prepared CdS with special microflower configuration consists of several petaloid nanosheets and the thickness of the freestanding single ultrathin nanosheets is about 20 nm (the inset of Fig. 1b). EDS data (Fig. 1e) confirms that the CdS microflowers only have two kind of elements, namely cadmium and sulphur and their atomic ratio is close to 1 : 1. The EDS surface scanning images prove that the Cd^2+^ and S^2−^ are distributed uniformly in CdS nanomaterials (as shown in Fig. 1c). XRD analysis of CdS-3 (Fig. 1d) shows that all the diffraction peaks are consistent with the standard data of CdS (JCPDS no. 1-780). This result further confirms that the as-obtained CdS nanomaterials has pure-phase. The XRD diffraction peaks of pure CdS located at 25.0°, 26.6°, 28.4°, 36.65°, 43.68°, 47.84° and 51.91° are corresponding to (1 0 0), (0 0 2), (1 0 1), (1 0 2), (1 1 0), (1 0 3) and (1 1 2) planes of hexagonal phase, respectively. In addition, the sharp peaks of CdS-3 also demonstrate that the product has an advantageous growth condition and high crystallinity. According to the analysis of XRD data, drawn the crystalline structure of CdS (Fig. 1f). The interlayer spacing between 2.52 Å to 4.20 Å and considerably larger than Cd ion (*d* = 1.97 Å) and Cu ion (*d* Cu(i) = 1.54 Å, *d* Cu(ii) = 1.46 Å).

To further investigate the surface chemical states of CdS, the binding energies of elements were measured by X-ray photoelectron spectroscopy (XPS). As indicated in Fig. 1g, only the peaks of O, C, S and Cd reveal in the XPS spectra of CdS nanostructures, and no other obvious elements are observed, further indicating the high purity of the CdS nanostructures. Fig. 1h presents the XPS spectra of Cd 3d orbit, and two main peaks of Cd 3d region are related to Cd 3d_5/2_ and Cd 3d_3/2_, respectively with the binding energy located at 404.58 and 411.34 eV, which is similar to those reported in the literature.^37,38^Fig. 1i shows the core level spectra of S 2p region, and the two peaks located at 160.78 and 161.79 eV are attributed to S 2p_3/2_ and S 2p_1/2_, respectively. The peaks in Fig. 1h and i are indicative of typical Cd and S of CdS. In addition, the binding energy concentrated in the range of 166–170 eV matches S–O bond resulted from the oxidation effect.^39^

A series of time-dependent experiments were performed to explore the growth mechanism of the 3D hierarchical CdS microflowers. Fig. S1a–c† shows the SEM images of CdS-1, CdS-2 and CdS-3 obtained at 160 °C for different times. In order to clarify the growth process of the 3D hierarchical CdS microflowers, the simulation diagram of morphology evolution process is shown in Fig. S1.† During the early stages of the reaction, the Cd^2+^ and S^2−^ are combined rapidly and generated crystal nucleons in solution. The SEM image of CdS-1 shows that the collected precipitation is composed of some irregular particles. As the reaction proceed, some nanosheets gradually grow on these nanoparticles (as shown in the Fig. S1b†). Finally, the SEM image of CdS-3 shows these nanosheets grow up into lily-like microflowers when the reaction time is 4 h. For the vivid description of the growth mechanism, the nucleons of crystals are likened to seeds. In the second stage, some nanosheets gradually grow on the crystal nucleus just like the process of seed germination. The buds eventually grow up into microflowers could be regard as the last stage of crystal growth mechanism. While in the growth of the lily-like CdS microflowers, the CTAB surfactant plays a critical role. Fig. S1† vividly shows the role of action of CTAB micelles.^43^ As a kind of cationic surfactant, CTAB micelles could vary the shape by changing the polarity of the solvent and the concentration. When the surfactant concentration is above the second critical micelle concentration,^40–42^ the micelles exist in lamellar type. The hydrophilic group outwards of laminar micelles would attract free Cd^2+^ in the precursor solution because of the coordination interaction and the Cd^2+^ are orderly arranged on the outer edge of the micelles. Meanwhile, the Cd^2+^ would attract free S^2−^ in solution. Therefore, CdS is orderly growth into sheets along the micellar lateral. In the growth process, the role of action of CTAB surfactants have similar function with the soft-template and induce the formation of the microflowers structure.^44,45^ Because the structure-directing effect of the CTAB template agent could optimize the microstructure of the semiconductors, the as-grown hierarchical CdS microflowers show large surface area and abundant active sites, possessing a potential application prospect. Moreover, the lily-like CdS microflowers could be used as a structural template to prepare other nanocrystals *via* insertion and exchange of atoms.^24,36^

**Fig. 2:**
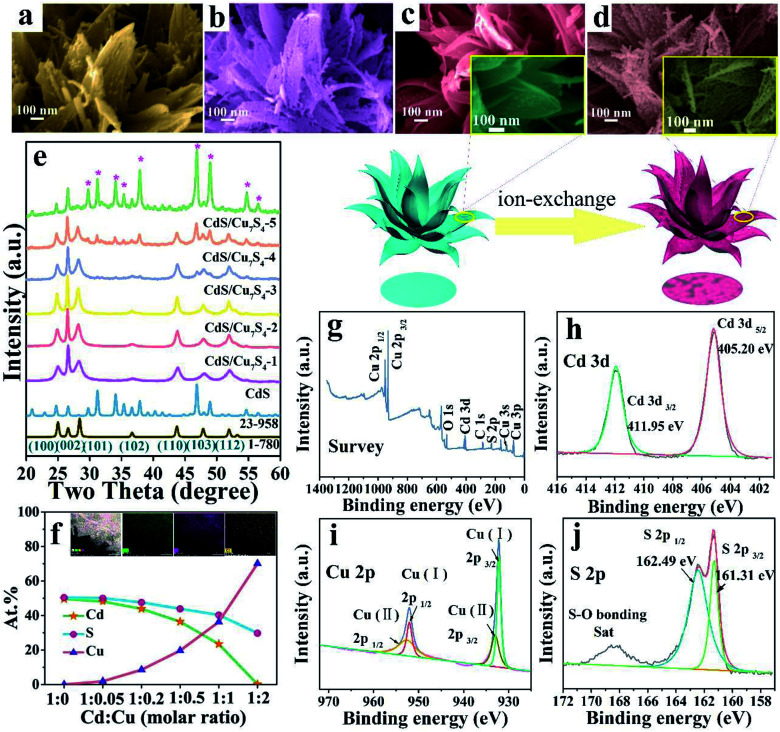
The schematic diagram of cation exchange reaction. SEM images of CdS/Cu_7_S_4_-3 (a) and CdS/Cu_7_S_4_-4 (b); SEM images in low and high magnification (inset) of CdS (c), CdS/Cu_7_S_4_-5 (d); (e) XRD patterns of different samples; (f) the curve graph of Cu, Cd and S elements content in the different samples and the EDS surface scanning images (inset) of CdS/Cu_7_S_4_-5; XPS spectra showing the (g) survey spectrum, (h) Cd 3d, (i) Cu 2p and (j) S 2p peak regions of CdS/Cu_7_S_4_-5.

In order to further improve the performance of the materials and maintain the special morphology, CdS/Cu_7_S_4_ nanocrystals are synthesized *via* partial and entire copper-cation exchange into CdS. Fig. 2a–d, S2c and d† show that the CdS/Cu_7_S_4_ completely inherit the framework from parent material. CdS/Cu_7_S_4_-5 nanocrystal maintained the morphology of the original template materials perfectly, while analysis from microstructure, from CdS to Cu_7_S_4_, small particles and holes to replace the original smooth complete nanosheets. This change is attribute to the crystalline phase transition from hexagonal to monoclinic after the cation exchange reaction, different lattice parameters caused the formation of crystal defects. In addition, the influence of reaction temperature on the morphology of the product has been explored (Fig. S3†) and the microstructures of products are collapsed at the higher reaction temperature. Morphology change of the nanocrystals, especially when the crystal phase changed before and after reaction, can be an important issue when the initial nanocrystals are used as the structural template.^46^ The crystalline phase could be confirmed by combing X-ray powder diffraction (Fig. 2e). Compared with Cu_1.97_S nanocrystals, the as-prepared CdS/Cu_7_S_4_-5 microflowers has higher BET surface areas and larger pore size (Fig. S4†).

The XRD patterns clearly demonstrated that the CdS/Cu_7_S_4_-1, CdS/Cu_7_S_4_-2, CdS/Cu_7_S_4_-3 and CdS/Cu_7_S_4_-4 contain two components, that is, CdS (JCPDS no. 1-780) and Cu_7_S_4_ (JCPDS no. 23-958), respectively (Fig. 2e). Due to the small amount of Cd contained in CdS/Cu_7_S_4_-5, there is no obvious characteristic peak of CdS in the XRD patterns. The XRD diffraction peaks of pure Cu_7_S_4_ located at 22.92°, 24.72°, 26.60°, 29.76°, 31.22°, 34.08°, 35.35°, 46.86° and 48.92° could be indexed as (8 6 1), (3 7 2), (6 0 0), (8 0 4), (8 2 1), (20 0 1), (20 4 0), (0 16 0) and (8 8 6) planes of monoclinic phase, indicating that the phase is changed from hexagonal phase to monoclinic phase after the ion exchange reaction. According to the above analysis, the Cu_7_S_4_ phase is generated gradually as the copper ion doping. Additionally, the energy-dispersive X-ray (EDX) maps of CdS/Cu_7_S_4_-4 (Fig. S5†) clearly reflect the element distribution of Cu, Cd and S atoms when the molar ratio of Cd^2+^ and Cu^2+^ is 1 : 1. With the increase of doped copper ions, the characteristic peaks of Cu_7_S_4_ are emerged gradually which can also be proved by the XRD patterns. The element content analysis of EDX (Fig. 2f) shows that the ratio of Cu : S contents of CdS/Cu_7_S_4_-5 is 70.15 : 29.66, while the cadmium atom content in CdS/Cu_7_S_4_-5 is just 0.19. It is suggested that the most of CdS phase is transformed into Cu_7_S_4_ when doped copper ion is superfluous. The inset of Fig. 2f shows the energy-dispersive X-ray (EDX) maps of CdS/Cu_7_S_4_-5, which exhibits that Cu, Cd and S elements show homogeneous distribution, further revealing that the heterojunction materials of Cu_7_S_4_ nanostructures and CdS QDs are prepared successfully by *in situ* ion exchange reaction.

In order to investigate the valence state and chemical composition, the CdS/Cu_7_S_4_-5 nanostructures were further conducted with X-ray photoelectron spectroscopy (XPS) analysis. As indicated in Fig. 2g, only the peaks of O 1s, C 1s and S 2p appear along with Cu 2p and Cd 3d in the XPS surveys of CdS/Cu_7_S_4_-5 nanostructures. Fig. 2h presents the XPS spectra of Cd 3d orbit, and two main peaks of Cd 3d region are related to Cd 3d_5/2_ and Cd 3d_3/2_, respectively with the binding energy located at 405.20 eV and 411.95 eV. Fig. 2i presents the XPS spectra of Cu 2p orbit, and two core level peaks of Cu 2p region located at 932.32 and 952.15 eV are corresponding to Cu 2p_3/2_ and Cu 2p_1/2_, respectively, which proves the presence of Cu^+^ in the CdS/Cu_7_S_4_-5 nanostructures. And the two peaks located at 933.53 and 953.03 eV are assigned to Cu 2p_3/2_ and Cu 2p_1/2_, respectively, demonstrating the existence of Cu^2+^ in the CdS/Cu_7_S_4_-5 nanomaterials. Fig. 2j shows the core level spectrum of the S 2p region, in which the binding energies of 161.31 and 162.49 eV are attributed to S 2p_3/2_ and S 2p_1/2_, respectively, indicating the existence of S^2−^ coordinated with Cu^+^ and Cu^2+^ species in the CdS/Cu_7_S_4_-5 nanostructures. In addition, the binding energy concentrated in the range of 168–170 eV matches with S–O bond resulted from the oxidation effect.^47,48^

**Fig. 3:**
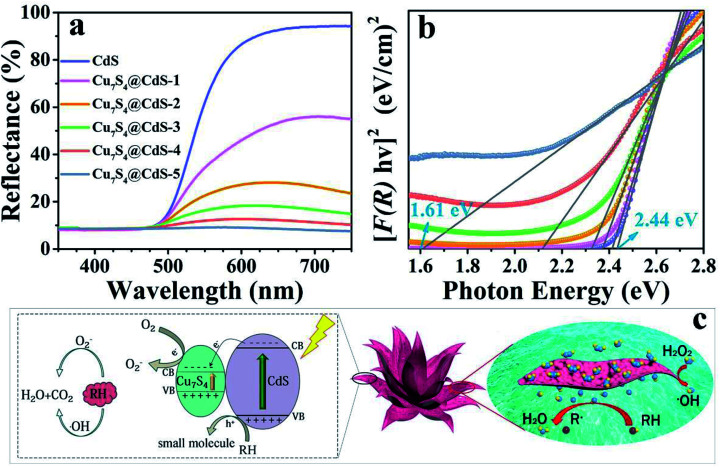
(a) UV-vis diffuse reflectance spectra for the CdS and CdS/Cu_7_S_4_ nanomaterials, (b) Kubelka–Munk-transformed diffuse reflectance spectra for the CdS and CdS/Cu_7_S_4_ nanomaterials used for the estimation of direct band gap, (c) the synergistic catalytic mechanism diagram of the CdS/Cu_7_S_4_ catalysts.

The diffuse reflectance spectra of the CdS and CdS/Cu_7_S_4_ nanomaterials were obtained through UV-vis absorbance spectroscopy (Fig. 3a). A sharp decrease in reflectance started at around 600 nm for the CdS nanomaterial due to strong absorption and the absorption edge of CdS/Cu_7_S_4_-1, CdS/Cu_7_S_4_-2, CdS/Cu_7_S_4_-3, CdS/Cu_7_S_4_-4 and CdS/Cu_7_S_4_-5 were suffered a gradual redshift. The diffuse reflectance spectra curve of the CdS/Cu_7_S_4_-5 nanomaterial was close to a flat line in the wavelength of 350 to 750 nm, indicating the strong absorption ability of the sample. The direct band gap of the samples could be evaluated by a Kubelka–Munk-function as eqn (1):^49^

1 *F*(*R*_∞_) = (1 − *R*_∞_)^2^/2*R*_∞_

where *R*_∞_ is the reflectance of an infinitely thick sample with at each wavelength (a material with *R*_∞_ ≈ 1, such as barium sulfate, is usually used as a laminate material). Therefore, plugging *R* into the above function and the [*F*(*R*)*hv*]^2^*vs. hv* plot was obtained (Fig. 3b). Bandgap energy of the CdS, CdS/Cu_7_S_4_-1, CdS/Cu_7_S_4_-2, CdS/Cu_7_S_4_-3, CdS/Cu_7_S_4_-4 and CdS/Cu_7_S_4_-5 nanomaterials were evaluated from the [*F*(*R*)*hv*]^2^*vs. hv* plots, indicating that the direct band gap value of CdS was 2.44 eV, CdS/Cu_7_S_4_-1 was 2.41 eV, CdS/Cu_7_S_4_-2 was 2.37 eV, CdS/Cu_7_S_4_-3 was 2.32 eV, CdS/Cu_7_S_4_-4 was 2.12 eV and CdS/Cu_7_S_4_-5 was 1.61 eV, consistent with the absorption edge of the corresponding samples. The evaluated direct band gap value of CdS was very close to the reported.^50,51^ Contrast CdS and CdS/Cu_7_S_4_-5, the band gap value was tuned from about 2.44 eV to 1.61 eV and the narrow band gap is conducive to the generation of photogenerated electron–hole pairs, thus improving the photocatalytic performance.

To better understand the effect of Cu ion-exchange on the band structure of the as-prepared catalysts, the Mott–Schottky test was performed in 0.2 M Na_2_SO_4_ aqueous solution at pH 7.^52,53^ As shown in Fig. S6,† the estimated band potentials of the samples are −0.61, −0.59, −0.59, −0.56, −0.62 and −0.60 eV *versus* a saturated calomel electrode (SCE) for CdS, CdS/Cu_7_S_4_-1, CdS/Cu_7_S_4_-2, CdS/Cu_7_S_4_-3, CdS/Cu_7_S_4_-4 and CdS/Cu_7_S_4_-5, respectively. The CB potentials *vs.* SCE were converted to the normal hydrogen electrode (NHE) scale by *E*_NHE_ = *E*_SCE_ + 0.197 eV at pH 7, and the converted values are −0.413, −0.393, −0.393, −0.363, −0.423 and −0.403 eV *versus* NHE for CdS, CdS/Cu_7_S_4_-1, CdS/Cu_7_S_4_-2, CdS/Cu_7_S_4_-3, CdS/Cu_7_S_4_-4 and CdS/Cu_7_S_4_-5, respectively. It is obvious that the band gap and potential of VB both change significantly upon Cu ion-exchange. Photoluminescence (PL) spectra were used to investigate the properties of the all samples at room temperature. As shown in Fig. S7,† with an excitation wavelength at 450 nm, all the nanostructures exhibit similar PL emission peaks at ∼556 nm, however, with the increase of Cu ion content, the peak intensity gradually weakened. The decrease of PL peak intensity indicates that the recombination rate of photogenerated electron–hole decreases, which leads to the increase of the efficiency of carriers participating in the photocatalytic process. To further understand the charge separation and transfer efficiency of the photogenerated carriers in the semiconductor materials, as shown in Fig. S7,† the photocurrent experiments were performed in a three-electrode photoelectrochemical cell with multiple 40 s on–off cycles. While the photocurrent density of the CdS/Cu_7_S_4_-5 is significantly higher than other catalysts, indicating that higher separation efficiency of the photogenerated electron–hole pairs and lower recombination rate in the CdS/Cu_7_S_4_-5 photocatalyst under the visible-light illumination.

The as-prepared 3D hierarchical lily-like CdS/Cu_7_S_4_ microflowers with high specific surface area and adequate surface active sites were effectively used as the catalysts for the degradation of organic dye such as MB and RB molecules. As show in Fig. 3c, the degradation mechanism of organic contaminants over the CdS and CdS/Cu_7_S_4_ was further explored. In an environment of complete darkness, the degradation of MB and RB mainly depends on the hydroxyl radical produced by hydrogen peroxide decomposition under the catalysis of copper ion, then these hydroxyl radicals would degrade the organic molecules into smaller molecules (H_2_O and CO_2_). The following are the relevant equations of the degradation reaction:

2 Cu^2+^ + H_2_O_2_ → H^+^ + CuOOH^+^

3 CuOOH^+^ → HOO˙ + Cu^+^

4 Cu^+^ + H_2_O_2_ → Cu^2+^ + OH^−^ + ·OH

5 RH + ·OH → small molecules

The Cu^2+^ react with H_2_O_2_ to promote the formation of hydroxyl radicals and the generated Cu^+^ are oxidized into Cu^2+^ by H_2_O_2_ (eqn (2)–(4)). The combination of the Cu^2+^ and H_2_O_2_ is known as Fenton-like reagent. The degradation rate of the organic dye solution and the formation rate of the hydroxyl radicals are positively related. Thus, after the introduction of Cu^2+^ ions, the degradation rate increases enormously because the presence of Cu^2+^ ions accelerates the decomposition of H_2_O_2_ to release hydroxyl radicals to effectively degrade the organic molecules (eqn (5)).

While Fenton-like catalysis and photocatalysis occur simultaneously under the excitation of UV-vis light, the photo-excited electron on the VB (valence band) of CdS and Cu_7_S_4_ can be promoted to their own CB (conduction band) (eqn (6)), then the electrons from the CB of CdS to the CB of Cu_7_S_4_ while holes were still stranded in the VB of CdS, suppressing the recombination between photogenerated electrons and holes efficiency. The separated electrons respectively react with H_2_O_2_ and oxygen to generate hydroxyl radicals and superoxide radicals (eqn (7) and (8)), these radicals with high oxidation potential decompose organic molecules into H_2_O_2_ and CO_2_ (eqn (9)–(11)). In order to identify the major active radical in the degradation of MB solution,^54–56^ Fig. S8† shows that the degradation rate of MB solution with 1 mM of Na_2_-EDTA is the slowest, indicating that photogenerated holes are the major active radical in the degradation of MB solution. When IPA exists, the degradation rate of MB solution increases slightly, due to light and catalyst-induced decomposition of H_2_O_2_ to produce a large number of hydroxyl radicals play a second important role in the degradation of MB solution. However, the presence of benzoquinone, the MB degradation rate is very close to that of scavengers free. The results show that the hole and ·OH are the main active radicals in the degradation of MB solution. The following are the relevant equations of the degradation reaction:

6 CdS/Cu_7_S_4_ → e^−^ + h^+^

7 O_2_ + e^−^ → ·O_2_^−^

8 H_2_O_2_ + e^−^ → ·OH + OH^−^

9 ·O_2_^−^ + RH → small molecules

10 ·OH + RH → small molecules

11 h^+^ + RH → small molecules

**Fig. 4:**
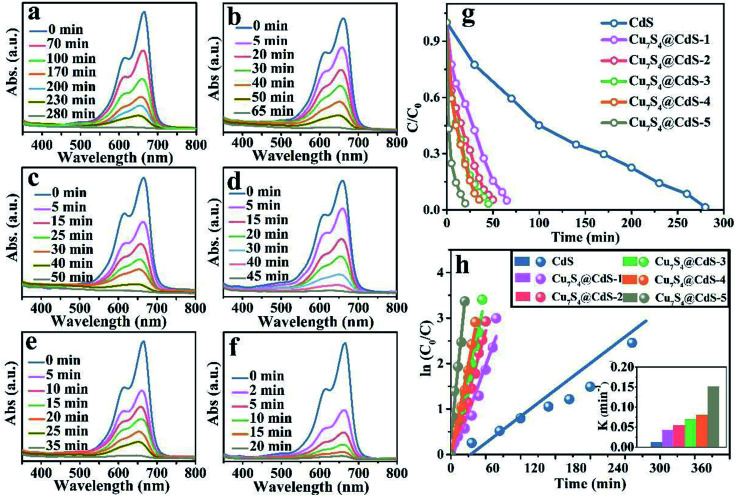
Time-dependent absorption spectra of catalytic degradation of MB under UV light using the following catalysts: (a) CdS, (b) CdS/Cu_7_S_4_-1, (c) CdS/Cu_7_S_4_-2, (d) CdS/Cu_7_S_4_-3, (e) CdS/Cu_7_S_4_-4, and (f) CdS/Cu_7_S_4_-5, (g) the degradation rate *vs.* irradiation time plots. (h) Kinetics study for the degradation of MB.

The degradation rate of H_2_O_2_ for the organic dye solutions without the assistance of the cocatalyst is very slow. With the deepening of the research, the ultraviolet light was introduced into the Fenton reaction and the oxidation performance of H_2_O_2_ was greatly improved. The degradation performance of as-prepared samples has been investigated by the UV-vis absorption spectra in Fig. 4a–f. All the experiments were executed by adding a certain amount of H_2_O_2_ and exposing to UV light for different times. Fig. 4a shows that after 280 min irradiation, the MB aqueous solution is degraded into almost colourless, which proved that the photocatalytic performance of pure CdS catalysts is unsatisfactory due to the ultrafast surface/bulk recombination of photogenerated charge carriers. The curves of the degradation of MB by CdS/Cu_7_S_4_ synthesized through cation exchange method are shown in Fig. 4b–e. It can be seen that the time for degrading the MB solution by the CdS/Cu_7_S_4_-1 is evidently reduced to about less than a quarter of the time which is needed by the pure CdS under the same conditions (as shown in Fig. 4b). This conclusion illustrates that the synergistic effect of photocatalytic and Fenton-like catalytic of CdS/Cu_7_S_4_ is evidently better than single photocatalytic of pure CdS catalyst.

Along with the increase of Cu_7_S_4_ components, the degradation time is gradually shortened. And the as-synthesized CdS/Cu_7_S_4_ materials from CdS/Cu_7_S_4_-1 to CdS/Cu_7_S_4_-5, spend 65 min, 50 min, 45 min, 35 min and 20 min to degrade MB solution to nearly 100%, respectively. The good catalytic performance is attributed to the special 3D hierarchical lily-like microstructure, the band gap adjustment and the synergistic effect of CdS/Cu_7_S_4_ catalysts. In order to further demonstrate the significant impact of structure and component has significant impact on the catalytic performance, the Cu_1.97_S nanomaterials with different morphology synthesized by a conventional method are used to compare with the as-prepared CdS/Cu_7_S_4_-5 microflowers (as shown in Fig. S2a, b and S9†). The obtained data shows that the directly synthesized Cu_1.97_S catalysts need 30 min to thoroughly degrade MB solution under the same conditions (as shown in Fig. S10†). Fig. 4g shows the degeneration curves for MB by the as-prepared samples. The decolouring degree are 15% for CdS, 44% for CdS/Cu_7_S_4_-1, 63% for CdS/Cu_7_S_4_-2, 74% for CdS/Cu_7_S_4_-3, 76% for CdS/Cu_7_S_4_-4 and 98% for CdS/Cu_7_S_4_-5 after the degradation of MB for 20 min under the same condition. The SEM images of CdS/Cu_7_S_4_-5 after the violent degradation reaction (Fig. S11†) show that it maintains the 3D hierarchical nanoflowers structure in general. To further study the degradation kinetics, the first order rate constant is calculated by eqn (12):

12 ln(*C*_0_/*C*) = *kt*

where *k* is the first order rate constant, *C*_0_ is the original concentration of MB solution, and *C* is the MB or RB@MB solution concentration at the specific reaction time. The first order rate constant of CdS, CdS/Cu_7_S_4_-1, CdS/Cu_7_S_4_-2, CdS/Cu_7_S_4_-3, CdS/Cu_7_S_4_-4 and CdS/Cu_7_S_4_-5 for degradation of the MB solution (Fig. 4h) are calculated to be 0.008 min^−1^, 0.039 min^−1^, 0.058 min^−1^, 0.076 min^−1^, 0.086 min^−1^ and 0.168 min^−1^, respectively.

**Fig. 5:**
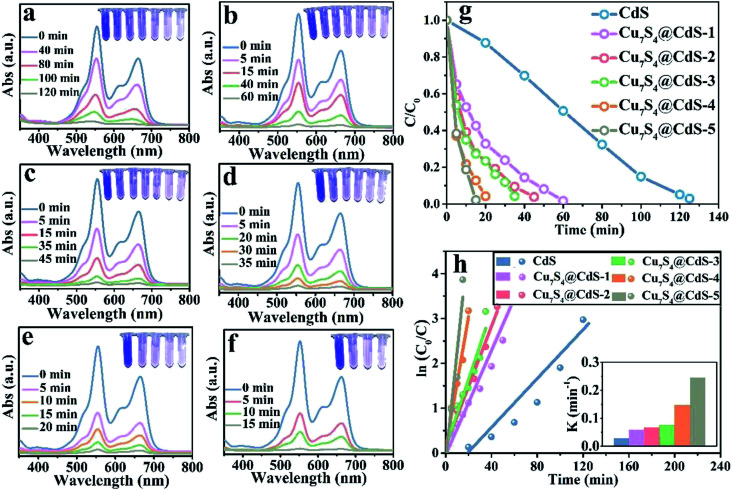
Time-dependent absorption spectra of catalytic degradation of RB@MB under UV light using the following catalysts: (a) CdS, (b) CdS/Cu_7_S_4_-1, (c) CdS/Cu_7_S_4_-2, (d) CdS/Cu_7_S_4_-3, (e) CdS/Cu_7_S_4_-4, and (f) CdS/Cu_7_S_4_-5, (g) the degradation rate *vs.* irradiation time plots, (h) kinetics study for the degradation of RB@MB.

The excellent performance degradation of the as-prepared samples not only for single organic dye, but also for the mixture of different kinds of organic dyes. The degradation performance for the RB@MB mixed solution of as-prepared samples has been investigated by the UV-vis absorption spectra in Fig. 5a–f. As shown in Fig. 5e, the decolouring degree are about 5% for CdS, 57.45% for CdS/Cu_7_S_4_-1, 72.34% for CdS/Cu_7_S_4_-2, 72.77% for CdS/Cu_7_S_4_-3, 87.23% for CdS/Cu_7_S_4_-4 and 97.87% for CdS/Cu_7_S_4_-5 after the degradation of MB for 15 min under the same condition. The directly synthesized Cu_1.97_S catalysts need 25 min to thoroughly degrade RB@MB solution under the same conditions (as shown in Fig. S10†). The first order rate constant of CdS, CdS/Cu_7_S_4_-1, CdS/Cu_7_S_4_-2, CdS/Cu_7_S_4_-3, CdS/Cu_7_S_4_-4 and CdS/Cu_7_S_4_-5 for degradation of the mixed solution of RB@MB (Fig. 5h) are calculated to be 0.027 min^−1^, 0.058 min^−1^, 0.067 min^−1^, 0.076 min^−1^, 0.148 min^−1^ and 0.245 min^−1^, respectively. It is clearly seen that the degradation rate of CdS/Cu_7_S_4_-5 is higher than that of other samples.

In addition, a series of contrast experiment is carried out in the case of a single variable. Fig. S12–S14† shows that the degradation capability of MB, RB, RB@MB under different illumination environment using CdS/Cu_7_S_4_-5 as catalyst and without catalyst. These test data reveal that in the absence of catalyst, the degradation performance is poor because of the decomposition of H_2_O_2_ become very slowly. When the visible light is used as the light source instead of the ultraviolet light, the catalytic performance is slightly weakened because of the weakening of the light energy. Even in the dark environment, the degradation capability is still substantial due to the high Fenton-like catalytic activity of the CdS/Cu_7_S_4_-5. Therefore, the ion exchange reaction can be used to modify the properties of crystalline materials and the synergistic catalytic effect of the as-prepared CdS/Cu_7_S_4_ nanocrystals has great practical and potential value in industrial wastewater treatment.

## Conclusions

In summary, ion exchange strategy was successfully popularized and applied to the construction of 3D hierarchical lily-like CdS/Cu_7_S_4_ QDs heterojunction material, owning the dual functions of photocatalyst and Fenton-like catalyst. During morphology genetic, the smooth nanosheets were transformed into the nanosheets composed of many nano particles, further endowing CdS/Cu_7_S_4_ QDs material with the larger specific surface area and more active sites. In the organic dyes degradation process, the degradation time of CdS/Cu_7_S_4_ QDs is about ten times shorter than CdS and robustly proved that the ion-exchange method is a feasible way to optimize the properties of nanocrystals.

## Conflicts of interest

There are no conflicts to declare.

## Supplementary Material

RA-009-C8RA09331H-s001
