# Supplementary material for: Bi-component synergic effect in lily-like CdS/Cu7S4 QDs for dye degradation
Source: RSC Adv. 2019 Jan 18;9(5):2441–50. doi: 10.1039/c8ra09331h (PMC9059895; doi:10.1039/c8ra09331h)
Supplement: RA-009-C8RA09331H-s001 [file RA-009-C8RA09331H-s001.pdf]

Electronic Supplementary Material (ESI) for RSC Advances.
This journal is © The Royal Society of Chemistry 2018

Supporting Information

Bi-component synergic effect in lily-like CdS/Cu_7S_4 QDs for dye degradation

Mengli Wan,[a,b] Shizhong Cui,[a] Wutao Wei,[a] Siwen Cui,[a] Kongyao Chen,[a] Weihua Chen,*[,b] and Liwei Mi,*[,a]

[a]Center for Advanced Materials Research, Zhongyuan University of Technology, Zhengzhou 450007, China.

[b]College of Chemistry and Molecular Engineering, Zhengzhou University, Zhengzhou 450001, China.

* Liwei Mi (a). E-mail: mlwzzu@163.com
* Weihua Chen (b). E-mail: chenweih@zzu.edu.cn

Synthesis of $Cu_{1.97}S$ nanomaterials by conventional method

$Cu_{1.97}S$ nanomaterials were prepared by a modified one step solvothermal method. In a typical procedure, 0.1812 g of copper nitrate trihydrate, 0.0571 g of thiourea, 0.1 g of CTAB, 9 ml of ethylene glycol and 15 ml of anhydrous ethylenediamine as the mixed solvent were successively added into a 30 ml of Teflon-lined autoclave, stirred for 0.5 h, maintained at 160 ^{o}C for 4 h and then cooled down to room temperature. The resulting dark solid products were collected by centrifugation and washed with distilled water and 95 % ethanol for several times. The product was then dried at 60 ^{o}C for 8 h, and collected for further characterization.

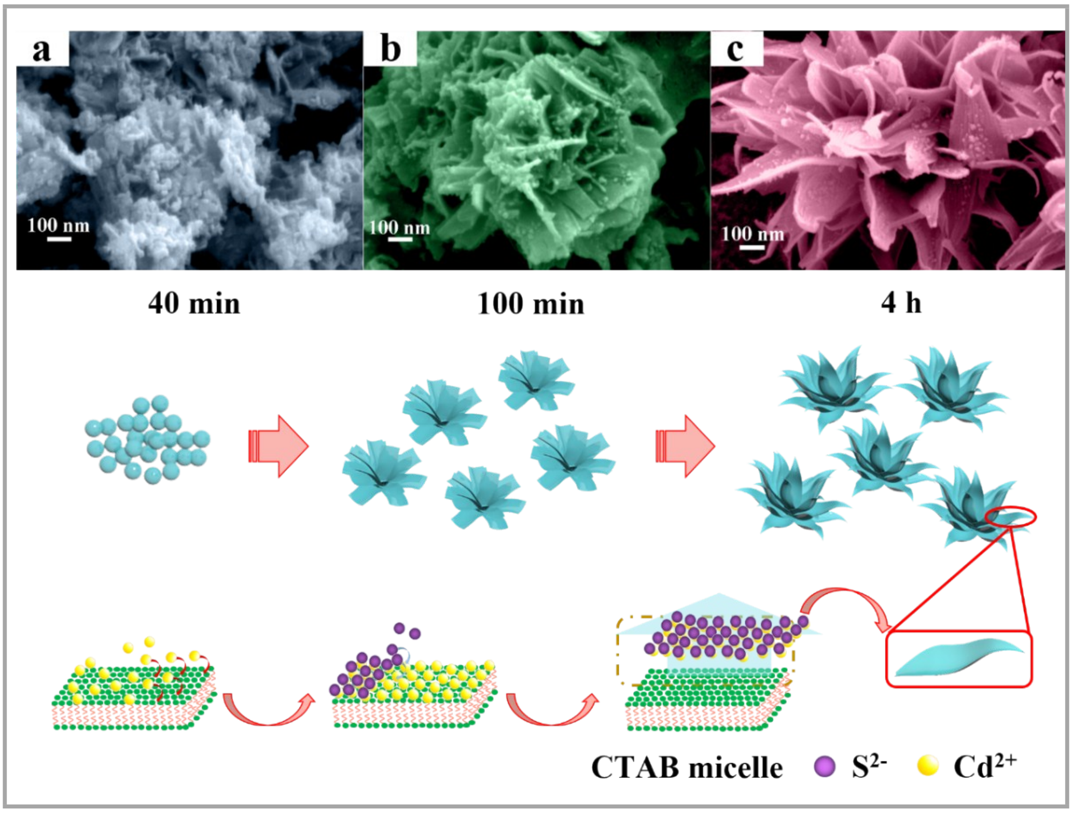

Figure S1. The SEM images of CdS nanomaterials prepared for 40 min (a), 100 min (b) and 4h (c) of reaction time. And the growth mechanism diagrams corresponding to different reaction time. The role of CTAB surfactant in CdS microflowers growth process.

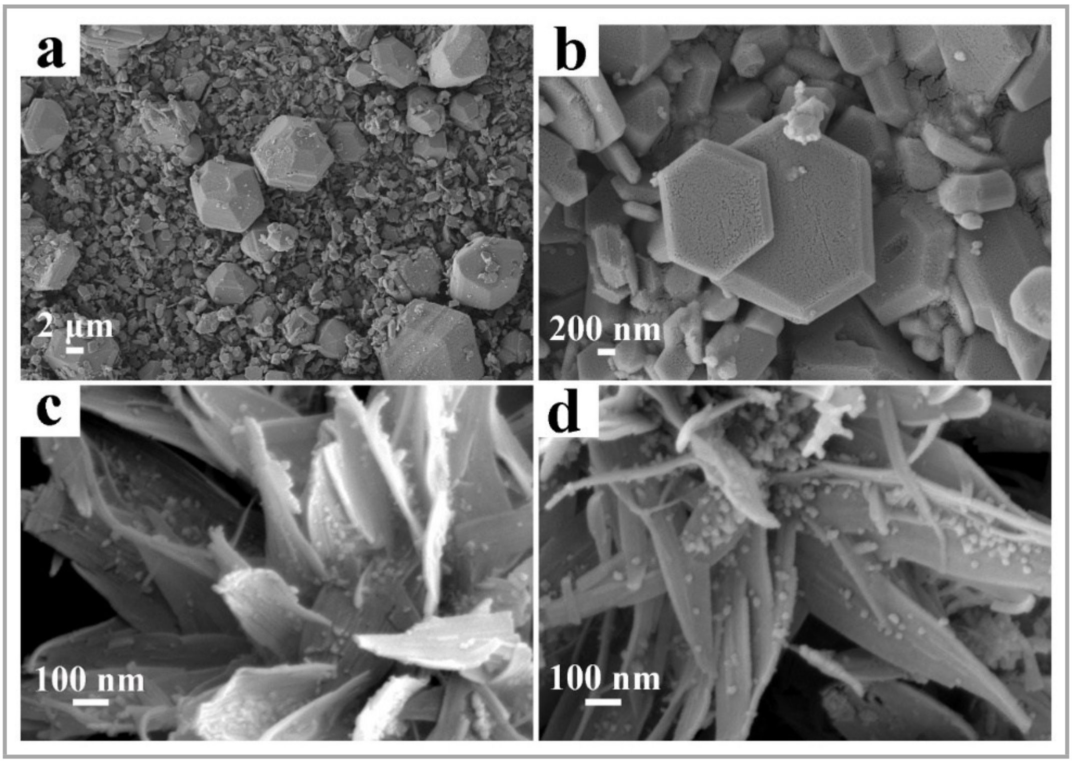

Figure S2. (a) (b) the SEM images of $Cu_{1.97}S$ nanomaterials.(c) the SEM image of CdS/Cu_7S_4-1, (d) the SEM image of CdS/Cu_7S_4-2

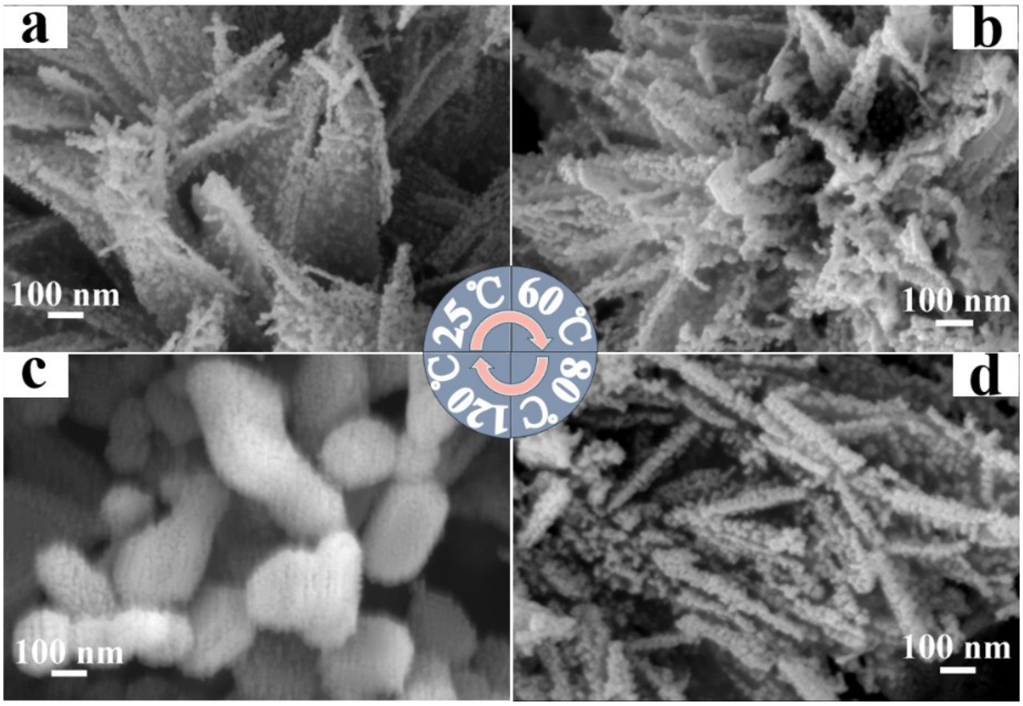

Figure S3. The SEM images of Cu_7S_4 prepared under different reaction temperature.

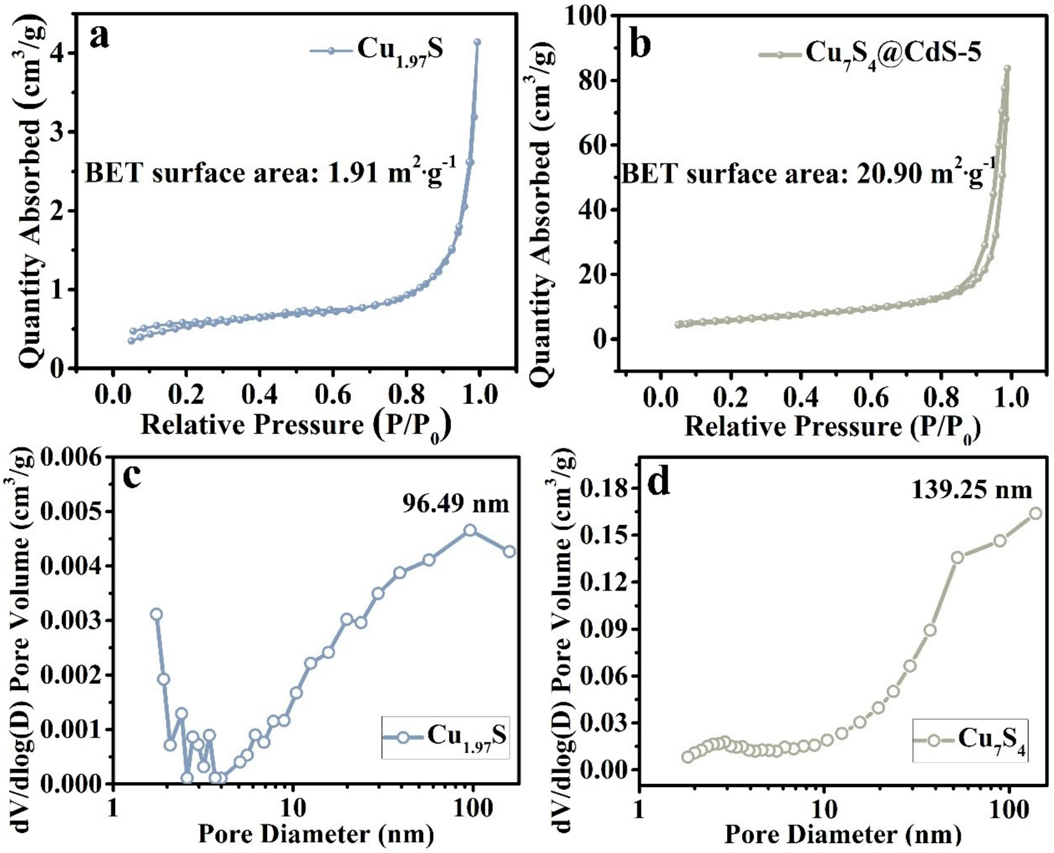

Figure S4. Nitrogen adsorption-desorption isotherm of (a) $Cu_{1.97}S$, (b) CdS/Cu_7S_4-5. And pore-size distribution of (c) $Cu_{1.97}S$, (d) CdS/Cu_7S_4-5.

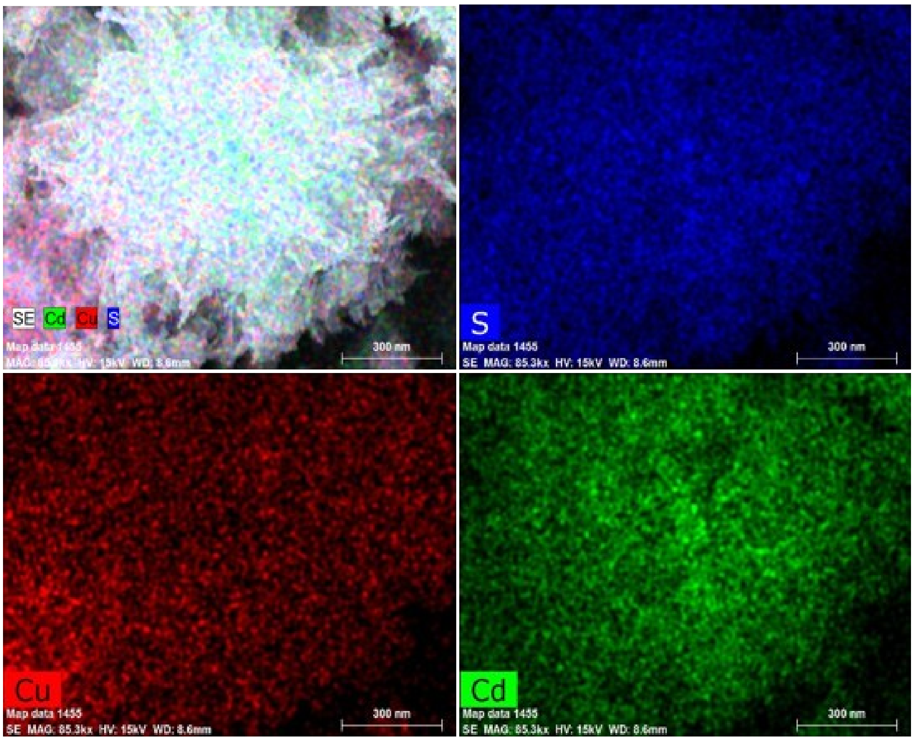

Figure S5. The EDS surface scanning images of CdS/Cu_7S_4 -4.

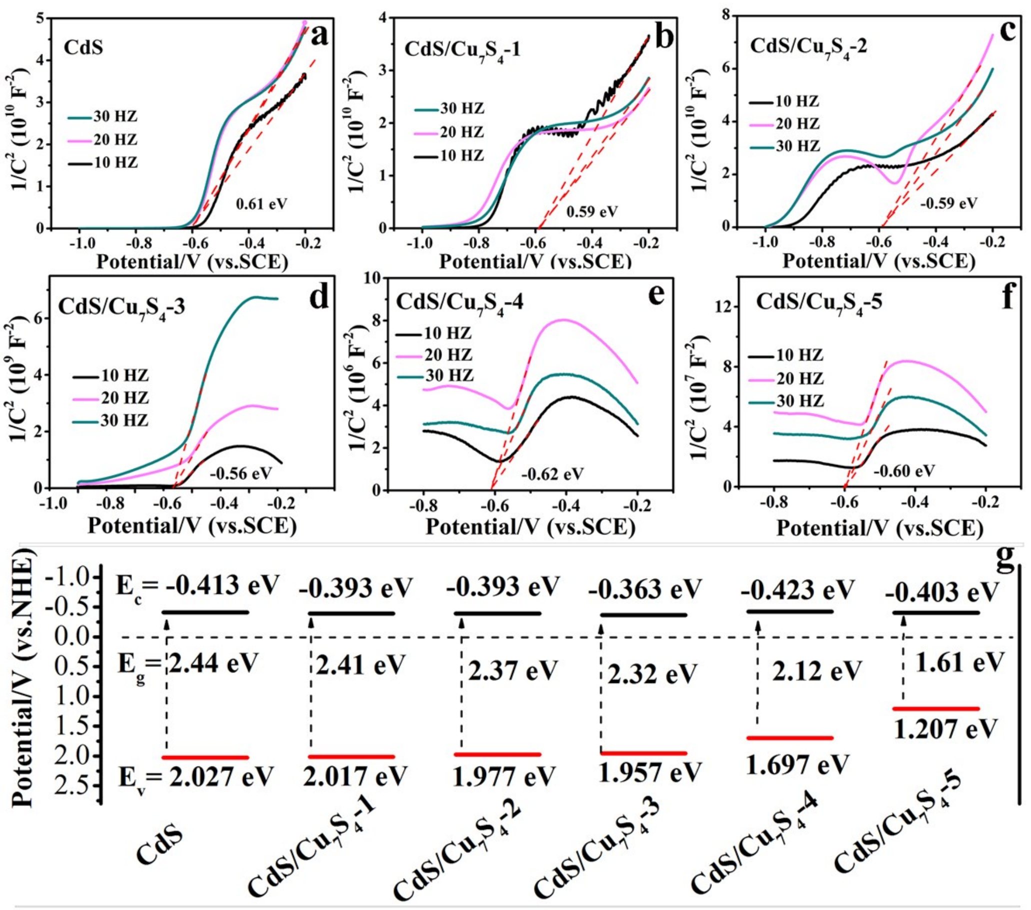

Figure S6. Mott-Schottky plots of the as-prepared samples: (a) CdS, (b) CdS/Cu_7S_4-1, (c) CdS/Cu_7S_4-2, (d) CdS/Cu_7S_4-3, (e) CdS/Cu_7S_4-4 and (f) CdS/Cu_7S_4-5. (g) VB and CB plots.

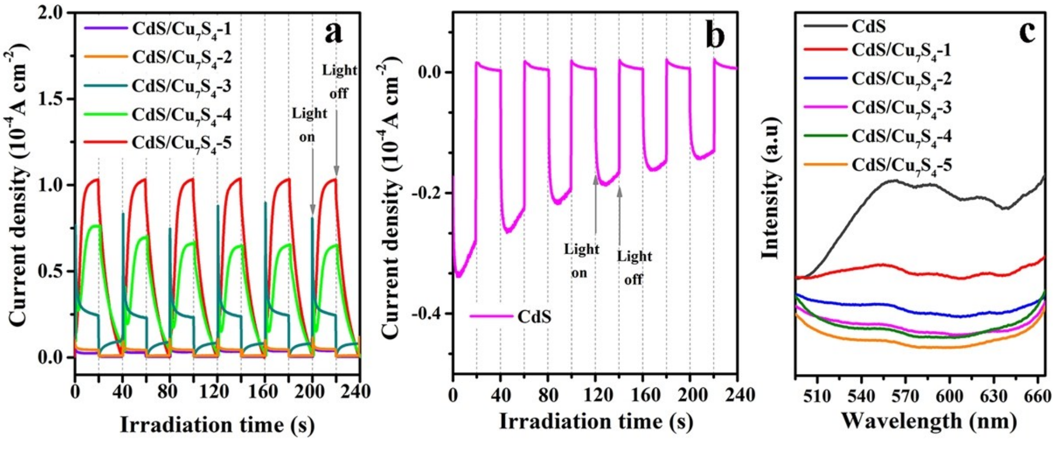

Figure S7. Photocurrent responses of the different samples under visible-light illumination: (a) the CdS/Cu_7S_4, (b) CdS. (c) Photoluminescence spectra of the different samples.

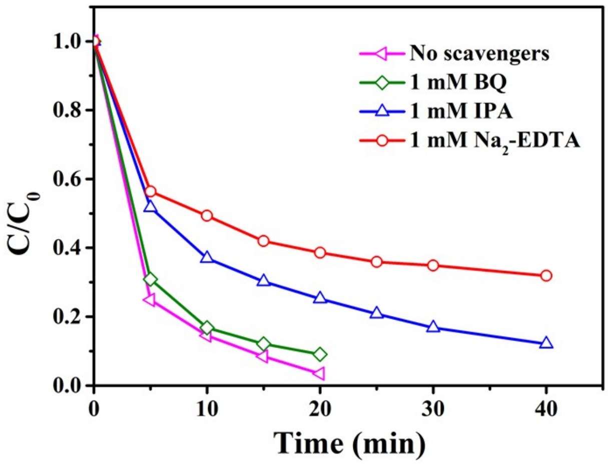

Figure S8. The effects of various scavengers for the photocatalytic degradation of MB solution using CdS/Cu_7S_4-5 photocatalyst.

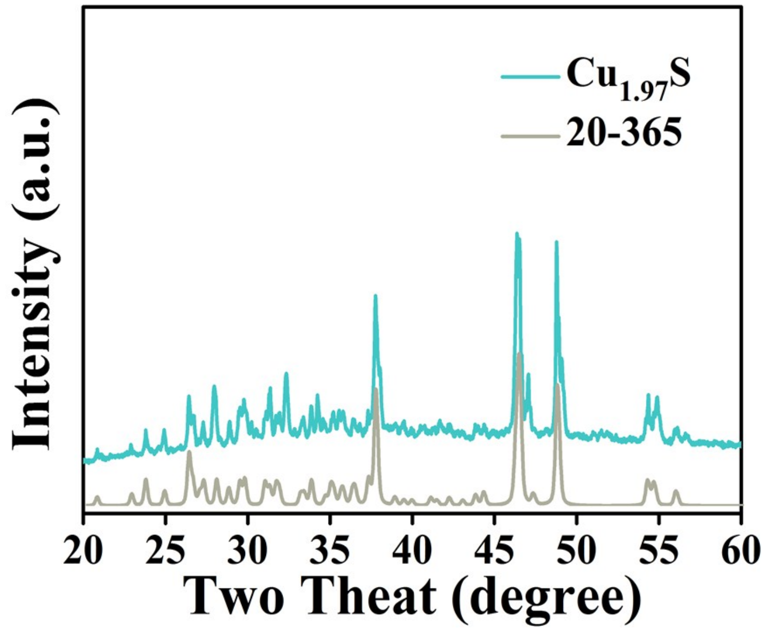

Figure S9. The XRD pattern of $Cu_{1.97}S$ nanomaterials.

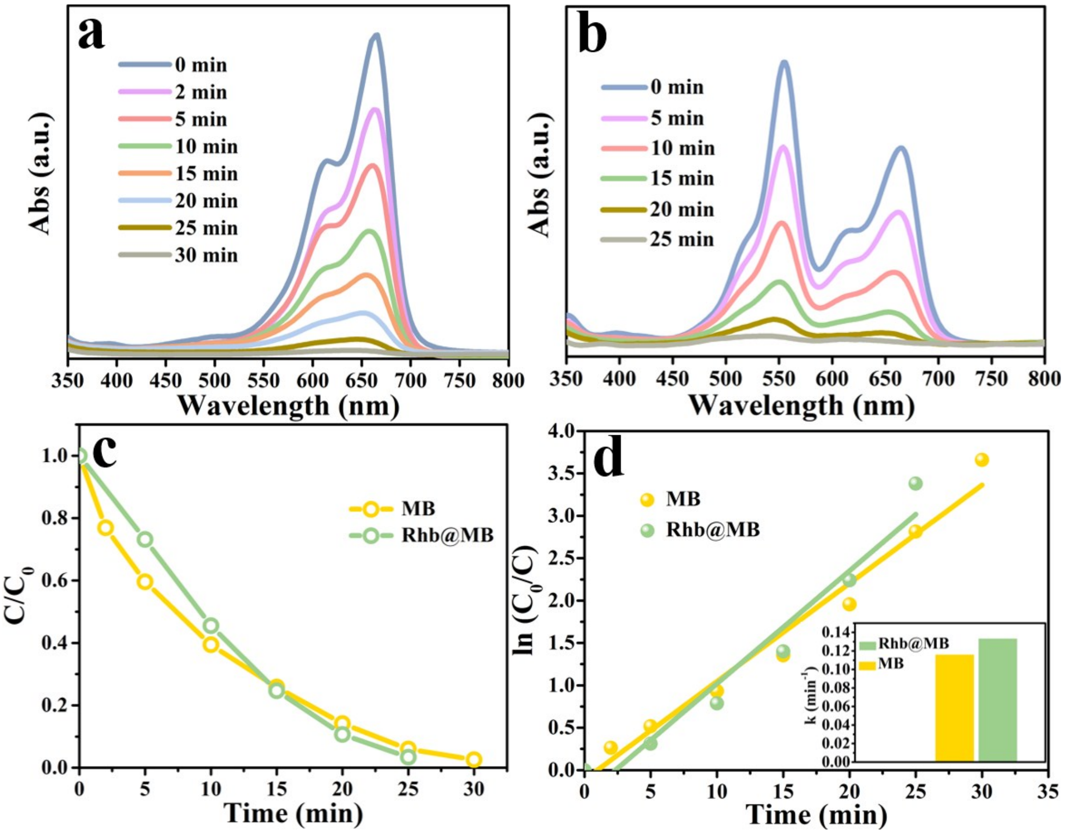

Figure S10. Time-dependent absorption spectra of degradation of (a) MB, (b) RB @ MB under UV light using $Cu_{1.97}S$ as catalysts, (c) The degradation rate vs. irradiation time plots, (d) Kinetics study for the degradation.

The degradation performance for the MB and RB@MB mixed solution of as-prepared $Cu_{1.97}S$ catalyst has been investigated by the UV-vis absorption spectra in **Figure S10.** As shown in **Figure S10c**, the decoloring degree are about 23.13 %, 40.38 %, 60.59 %, 74.16 %, 85.86 %, 94.00 % and 97.42 % after the degradation of MB for 2 min, 5 min, 10 min, 15 min, 20 min, 25 min and 30 min, respectively, and the decoloring degree are 26.81 %, 54.47 %, 75.32 %, 89.36 % and 96.60 % after the degradation of RB@MB for 5 min, 10 min, 15 min, 20 min and 25 min, respectively. The first order rate constant of $Cu_{1.97}S$ for degradation of the MB and RB@MB (**Figure S10d**) are calculated to be 0.1159 min^{-1} and 0.1331 min^{-1}, respectively.

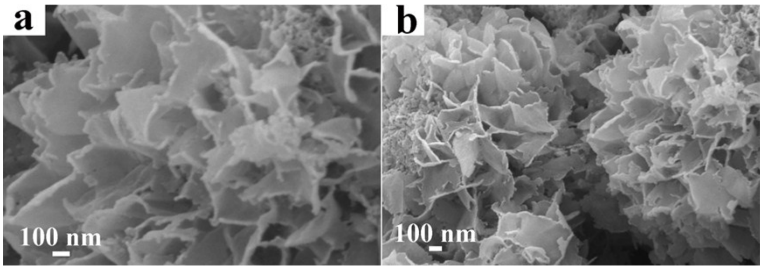

Figure S11. The SEM images of CdS/Cu_7S_4-5 after degradation reaction.

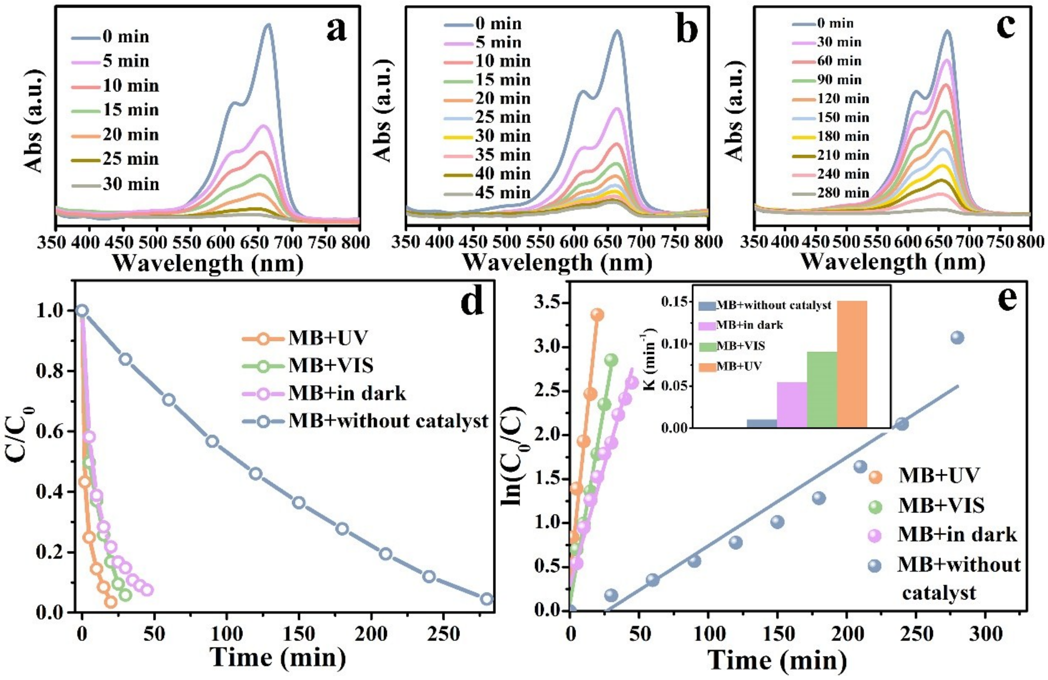

Figure S12. Time-dependent absorption spectra of catalytic degradation of MB (a) under visible light and (b) in dark environment using CdS/Cu_7S_4-5 as catalysts, (c) Time-dependent absorption spectra of catalytic degradation of MB under UV light without catalysts, (d) The degradation rate vs. irradiation time plots, (e) Kinetics study for the degradation of MB: the inset shows the first order rate constant for CdS/Cu_7S_4-5.

As shown in **Figure S12d**, the decoloring degree are about 50.30 %, 63.01 %, 74.28 %, 83.24 %, 90.46 % and 94.22 % after the degradation of MB under visible light for 5 min, 10 min, 15 min, 20 min, 25 min and 30 min, respectively. The decoloring degree are about 41.79 %, 61.19 %, 71.64 %, 78.21%, 83.28%, 85.22%, 89.25%, 91.04% and 92.54% after the degradation of MB in dark for 5 min, 10 min, 15 min, 20 min, 25 min, 30 min, 35 min, 40 min

and 45 min, respectively. The decoloring degree are about 16.12%, 29.55%, 43.28%, 54.03%, 63.58%, 72.24%, 80.60%, 88.06% and 95.52% after the degradation of MB under UV light without catalysts for 30 min, 60 min, 90 min, 120 min, 150 min, 180 min, 210 min, 240 min and 280 min, respectively. The first order rate constant for degradation of the MB under visible light, in dark and under UV light without catalysts (**Figure S12e**) are calculated to be 0.0903 min^{-1}, 0.0547 min^{-1} and 0.0101 min^{-1}, respectively.

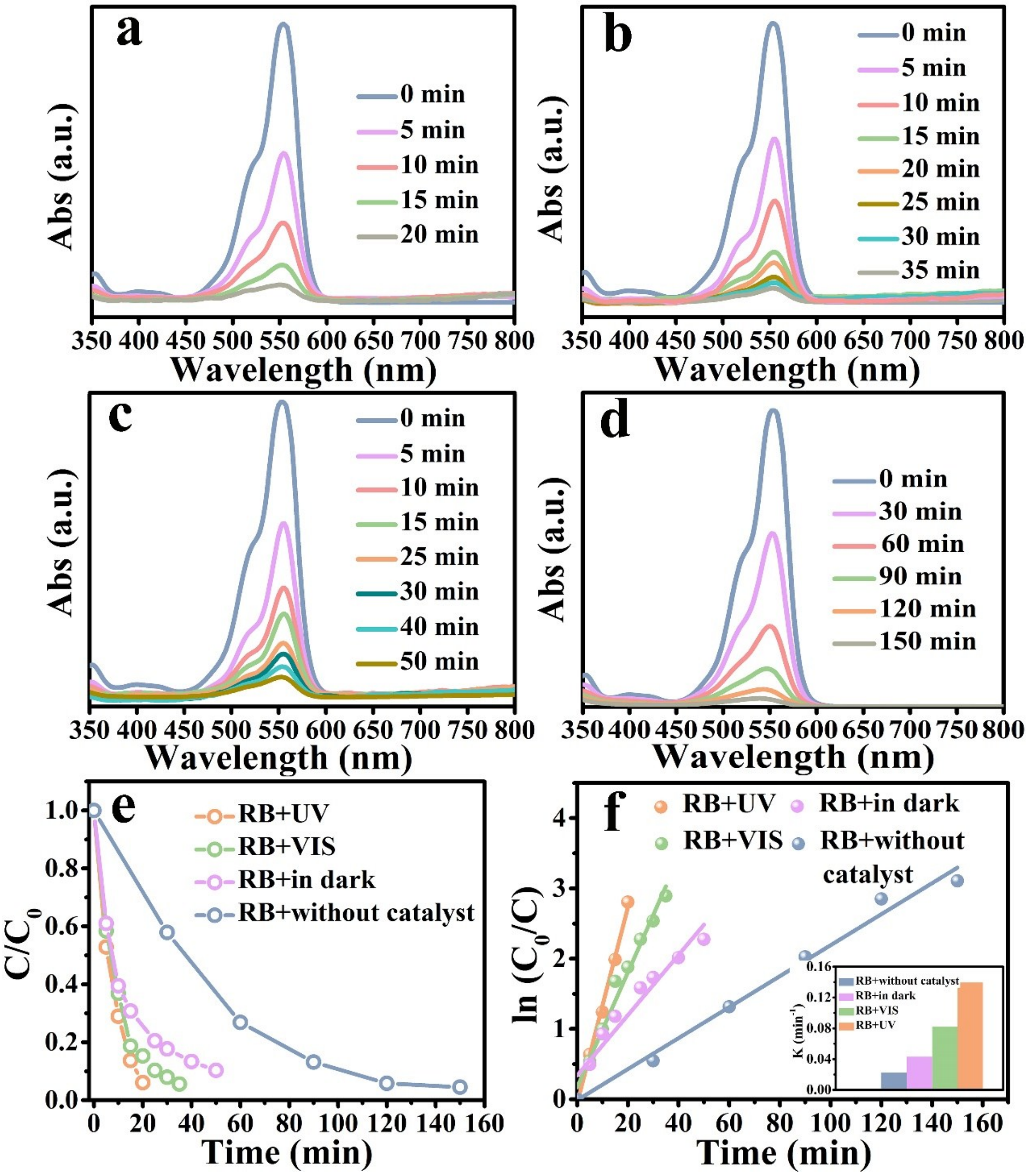

Figure S13. Time-dependent absorption spectra of catalytic degradation of RB (a) under UV light, (b) under visible light and (c) in dark environment using CdS/Cu_7S_4-5 as catalysts, (d)

Time-dependent absorption spectra of catalytic degradation of RB under UV light without catalysts, (e) The degradation rate vs. irradiation time plots, (f) Kinetics study for the degradation of RB: the inset shows the first order rate constant for CdS/Cu_7S_4-5.

As shown in **Figure S13e**, the decoloring degree are about 47.11 %, 71.05 %, 86.32 % and 93.95 % after the degradation of RB under UV light for 5 min, 10 min, 15 min and 20 min , respectively. The decoloring degree are about 41.58 %, 63.16 %, 81.32 %, 84.74 %, 89.74 %, 92.11 % and 94.47% after the degradation of RB under visible light for 5 min, 10 min, 15 min, 20 min, 25 min, 30 min and 35 min, respectively. The decoloring degree are about 38.97 %, 60.51 %, 69.23 %, 79.49 %, 82.31 %, 86.67 % and 89.74 % after the degradation of RB in dark for 5 min, 10 min, 15 min, 25 min, 30 min, 40 min and 50 min, respectively. The decoloring degree are about 42.11 %, 73.16 %, 86.84 %, 94.21 % and 95.52% after the degradation of RB under UV light without catalysts for 30 min, 60 min, 90 min, 120 min and 150 min, respectively. The first order rate constant for degradation of the RB under UV light, under visible light, in dark and under UV light without catalysts (**Figure S13f**) are calculated to be 0.1392 min^{-1}, 0.0817 min^{-1}, 0.0429 min^{-1} and 0.0221 min^{-1}, respectively.

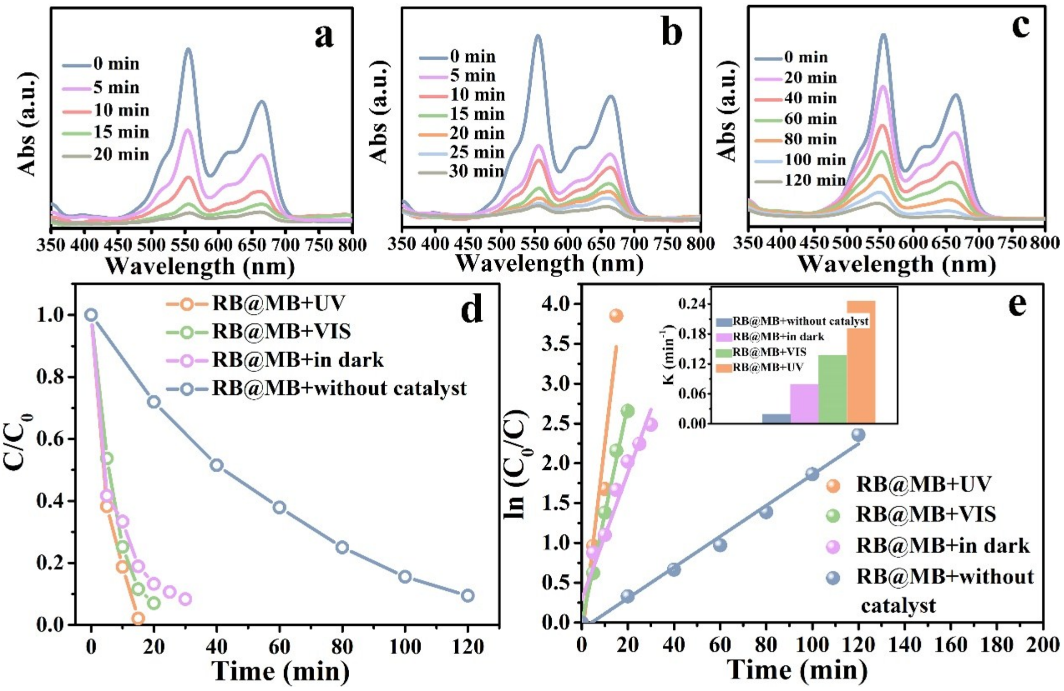

Figure S14. Time-dependent absorption spectra of catalytic degradation of RB@MB (a) under visible light and (b) in dark environment using CdS/Cu_7S_4-5 as catalysts, (c) Time-dependent absorption spectra of catalytic degradation of RB@MB under UV light without catalysts, (d) The degradation rate vs. irradiation time plots, (e) Kinetics study for the degradation of RB@MB: the inset shows the first order rate constant for CdS/Cu_7S_4-5.

As shown in **Figure S14d**, the decoloring degree are about 46.28 %, 74.79 %, 88.43 % and 92.98 % after the degradation of RB@MB under visible light for 5 min, 10 min, 15 min and 20 min, respectively. The decoloring degree are about 58.33 %, 66.67 %, 81.06 %, 86.74%, 89.40 % and 91.67 % after the degradation of RB@MB in dark for 5 min, 10 min, 15 min, 20 min, 25 min and 30 min, respectively. The decoloring degree are about 28.03 %, 48.48 %, 62.12 %, 75.00 %, 84.47 % and 90.53 % after the degradation of RB@MB under UV light without catalysts for 20 min, 40 min, 60 min, 80 min, 100 min and 120 min, respectively. The first order rate constant for degradation of the RB@MB under visible light, in dark and under UV light without catalysts (**Figure S14e**) are calculated to be 0.1369 min^{-1}, 0.0794 min^{-1} and 0.0194 min^{-1}, respectively.
